# Electroacupuncture Regulates Pain Transition Through Inhibiting PKCε and TRPV1 Expression in Dorsal Root Ganglion

**DOI:** 10.3389/fnins.2021.685715

**Published:** 2021-07-20

**Authors:** Junfan Fang, Sisi Wang, Jie Zhou, Xiaomei Shao, Haiju Sun, Yi Liang, Xiaofen He, Yongliang Jiang, Boyi Liu, Xiaoming Jin, Jianqiao Fang, Junying Du

**Affiliations:** ^1^Department of Neurobiology and Acupuncture Research, The Third Clinical Medical College, Zhejiang Chinese Medical University, Key Laboratory of Acupuncture and Neurology of Zhejiang Province, Hangzhou, China; ^2^Department of Anatomy, Cell Biology and Physiology, Stark Neuroscience Research Institute, Indiana University School of Medicine, Indianapolis, IN, United States

**Keywords:** electroacupuncture, hyperalgesic priming, dorsal root ganglion, protein kinase C epsilon, TRPV1

## Abstract

Many cases of acute pain can be resolved with few side effects. However, some cases of acute pain may persist beyond the time required for tissue injury recovery and transit to chronic pain, which is hard to treat. The mechanisms underlying pain transition are not entirely understood, and treatment strategies are lacking. In this study, the hyperalgesic priming model was established on rats to study pain transition by injection of carrageenan (Car) and prostaglandin E2 (PGE2). The expression levels of protein kinase C epsilon (PKCε) and transient receptor potential vanilloid 1 (TRPV1) in the L4–L6 dorsal root ganglion (DRG) were investigated. Electroacupuncture (EA) is a form of acupuncture in which a small electric current is passed between a pair of acupuncture needles. EA was administrated, and its effect on hyperalgesia and PKCε and TRPV1 expression was investigated. The PKCε–TRPV1 signaling pathway in DRG was implicated in the pain transition. EA increased the pain threshold of model animals and regulated the high expression of PKCε and TRPV1. Moreover, EA also regulated hyperalgesia and high TRPV1 expression induced by selective PKCε activation. We also found that EA partly increased chronic pain threshold, even though it was only administered between the Car and PGE2 injections. These findings suggested that EA could prevent the transition from acute to chronic pain by inhibiting the PKCε and TRPV1 expression in the peripheral nervous system.

## Introduction

Pain is a major health problem in the clinical practice. Fortunately, acute pain can be controlled and resolved through a variety of strategies with few psychological problems or side effects (Moore et al., 2015; Derry et al., 2016; Chou et al., 2017; Vilite et al., 2019). However, for some patients, the pain may persist beyond the time required for tissue injury recovery and transition to chronic pain. This type of pain usually lacks an obviously cause and has few effective treatment strategies (Marquie et al., 2003; van Wilgen and Keizer, 2012). The reasons underlying the failure to recover from acute pain are not yet understood.

In previous studies, a hyperalgesic priming model was developed to study the mechanisms underlying the transition from acute to chronic pain (Chen et al., 2014; Kim et al., 2016; Dai et al., 2017). It has been demonstrated that exposing the peripheral terminal of primary afferent nociceptors to inflammatory mediators [e.g., prostaglandin E2 (PGE2)] is able to induce long-lasting mechanical pain threshold that decreases depending on whether the terminal is pre-exposed to inflammatory mediators [e.g., carrageenan (Car)] (Kim et al., 2015; Megat et al., 2018). The long-lasting pain sensitization induced by PGE2 persists for more than 14 days without any obvious cause (Reichling and Levine, 2009), such as peripheral nerve injury, inflammation, or cancer. Previous studies have demonstrated that activating protein kinase C epsilon (PKCε) in the ipsilateral lumbar dorsal root ganglion (DRG) plays a pivotal role in pain transition (Ferrari et al., 2014; Kandasamy and Price, 2015). In addition, PKCε induces pain sensitization by activating its downstream protein (Ferrari et al., 2014). Transient receptor potential vanilloid 1 (TRPV1) is one of the downstream proteins of PKCε and is generally believed to be involved in the development and maintenance of various chronic pain (Malek et al., 2015). Furthermore, inhibiting PKCε activation in the DRG prevents the pain from transiting from acute to chronic in a hyperalgesic priming model (Parada et al., 2003). These results suggest preventing the transition from acute to chronic pain as a new possibility for the treatment of chronic pain.

However, PKCε not only is responsible for the pain transition but also contributes to various physiological functions (Chen and Tian, 2011; Kostyak et al., 2017). Its various roles present a particularly challenging problem, requiring clinicians to balance two competing interests: preventing the transition from acute to chronic pain and minimizing interference with physiological functions. One of the solutions is to inhibit PKCε expression or activation at a precise time point in a specific tissue or area, such as the DRG. These conditions are often challenging to achieve in practice. Another solution is to find methods that can regulate the transition from acute to chronic pain and can be applied with few side effects.

Electroacupuncture (EA) is a form of acupuncture in which a small electric current is passed between a pair of acupuncture needles. Many studies have demonstrated that EA can produce an analgesic effect (Hou et al., 2019; Xiang et al., 2019; Xu et al., 2019; Zheng et al., 2019). Our previous studies showed that EA alleviates mechanical hyperalgesia in both inflammatory and neuropathic pain models (Liang et al., 2019; Xiang et al., 2019). These effects are thought to be related to the interference of EA in DRG neuronal function or protein expression. In addition, EA is able to alleviate acute and chronic pain by activating the endogenous opioid peptide system (Cheng et al., 2013; Jiang et al., 2016). Based on its analgesic effect, EA may be a potential and few side-effects treatment option for preventing the transition from acute to chronic pain (Chai et al., 2018). Our previous study found that EA could regulate the pain threshold in a hyperalgesic priming model and down-regulate the expression level of PKCε in DRG (Wang et al., 2018, 2020). However, it is still not completely clear whether EA can intervene in the transition from acute to chronic pain or the mechanisms. Answering these related questions may provide new strategies for the treatment of chronic pain.

In the present study, we tested the role of the PKCε signaling pathway in the transition from acute to chronic pain in the peripheral nervous system and explored the potential role of EA in preventing pain transition using a rat hyperalgesic priming model.

## Materials and Methods

### Animals

Adult male Sprague–Dawley rats (weighing 180–230 g, 6–8 weeks) (animal certificate no. SCXK(Hu)2013-0016, Shanghai Laboratory Animal Center, Chinese Academy of Sciences) were used for this study. The animals were housed five per cage in the Laboratory Animal Center of Zhejiang Chinese Medical University (SYXK(Zhe)2013-0184) with food and water *ad libitum* in a controlled 12-h light/dark cycle environment (25°C ± 2°C, 50% ± 10%). All animal manipulation performed in this study complied with the institutional and governmental regulations regarding the ethical use of animals and was approved by the Experimental Animal Center Affiliated Zhejiang Chinese Medical University (approval no. IACUC-20180319-12).

### Drug Preparation

Carrageenan and PGE2 were purchased from Sigma-Aldrich (St. Louis, MO, United States). The PKCε agonist ψεRACK (peptide-sequence HDAPIGYD with a membrane permeable sequence) was synthesized by Bankpeptide (Hefei, China). The PKCε inhibitor PKCεV1-2 was purchased from Calbiochem, Milipore Sigma (Darmstadt, Germany). The TRPV1 antagonist capsazepine (CPZ) and AMG9810 were purchased from Sigma-Aldrich (St. Louis, MO, United States) and Abcam (United States). Morphine was purchased from Northeast Pharmaceutical Group Shenyang No.1 Pharmaceutical Co., Ltd. (Shenyang, China).

A stock solution of PGE2 (1 μg/μl) was prepared in 10% ethanol and dissolved in normal saline (NS) to a concentration of 100 ng/25 μl immediately before injection. Car was dissolved in NS to a concentration of 2% and stored. AMG9810 was prepared in Dimethyl sulfoxide as a stock and diluted in PBS to a concentration of 0.8 mg/25 μl before injection. The peptide ψεRACK, capsazepine, and PKCεV1-2 were prepared in NS to a concentration of 1 μg/25 μl immediately before injection. Morphine (10 mg/kg) was injected intraperitoneally.

The use of all the drugs in this study was based on previous studies.

### Hyperalgesic Priming Models

The hyperalgesic priming was induced as previously described (Kim et al., 2016). Rats were briefly anesthetized with 2.5% isoflurane to facilitate the intraplantar injection of Car, PGE2, or the other drugs used in this study. The injection site was first scrubbed with 75% alcohol. Hyperalgesic priming was induced by the intraplantar injection of 100 μl Car (first injection), and persistent hyperalgesia was induced by the injection of 25 μl of PGE2 (second injection) at 7 days after the first injection. In NS+PGE2 group rats, the same volume of NS instead of Car was administered.

### Drug Administration

Drugs were administered through intraplantar injection. The injection site was first scrubbed with 75% alcohol. In Car+ψεRACK and Car+ψεRACK+EA groups, selected PKCε agonist peptide ψεRACK instead of PGE2 was injected. In the Car+PGE2+PKCεV1-2 group, PKCεV1-2 was injected 5 min before PGE2 injection in this study. In the Car+PGE2+CPZ and Car+PGE2+AMG9810 group, CPZ and AMG9810 were injected 5 min before behavioral testing 48 h after PGE2 injection.

### Nociceptive Testing

Nociceptive behaviors were quantified before the first injection, 4 h, 24 h, 48 h, 72 h, and 7 days after the first injection, and 1, 4, 24, and 48 h after the second injection. Experimenters were blinded to the study condition for the duration of the experiment.

The mechanical withdrawal threshold (MWT) was tested using von Frey filaments (Stoelting Co., Wood Dale, IL, United States) by the up-down method as previously described (Chaplan et al., 1994). Von Frey filaments (0.4, 0.6, 1, 2, 4, 6, 8, 15, and 26 g) were pressed onto the lateral plantar surface of the ipsilateral paw. The first filament applied corresponded to a force of 2 g. A filament of a greater or lesser force was then chosen depending on whether the response was negative or positive. The responses were recorded as X or O. The results were calculated using a function previously described method (Chaplan et al., 1994).

The thermal withdrawal latency (TWL) was observed by using a laser machine (37370, UGO, Italy) as previously described. A constant intensity laser was applied to the plantar surface of the rat’s paw. The radiant heat was set at 35°C, and the cutoff time was set at 20 s to prevent injury. The latency time of the withdrawal response was automatically recorded by the instruments.

### EA Intervention

Five hours after the first injection, the EA intervention began following the completion of the behavioral test (Liang et al., 2019; Xiang et al., 2019). The rats were gently immobilized in a cotton retainer, with 5-mm-deep insertion of stainless-steel needles (0.18 mm × 13 mm) into the bilateral Zusanli (ST36, between the tibia and fibula 5 mm below the knee) and Kunlun (BL60, between the tip of the external malleolus and tendo calcaneus) acupoints. A series of EA intensities (0.5, 1.0, and 1.5 mA) was increased every 10 min by using a HANS Acupuncture Point Nerve Stimulator (LH-202H Huawei Co., Ltd., Beijing, China) connected to the needles. The EA persisted for 30 min once per day at 2/100 Hz (alternation of electrical stimulations at 2 Hz and 100 Hz every 3 s) until the end of the experiment or otherwise in designated specific cases. For the EA II group particularly, the EA intervention was administered once per day during the period between 5 h after the first injection and the second injection. Other parameters were consistent with the those described above.

The sham EA was administered subcutaneously into ST36 and BL60 of animals at a depth of 1 mm with the same needles connected to the same stimulator as described above, but without electrical stimulation.

Another sham EA was also administered. The same needles were inserted into the bilateral acupoints “Quchi” (LI 11, radial proximal anterior joint) and “Waiguan” (TE 5, 3 mm above the wrist) at a depth of 3 mm and connected with the HANS stimulator. The electrical stimulation was given as the same parameters describe above.

### Tissue Preparation

Animals were anesthetized with 2% pentobarbital sodium and sacrificed 4 and 48 h after the second injection when the MWT or TWL testing was completed. They were quickly perfused with 150 ml of NS (4°C). Then, DRGs was extracted rapidly and stored in a −80°C freezer for Western blotting experiments. For immunofluorescence, the animals were continuously perfused with 400 ml fresh 4% paraformaldehyde in 0.1 M phosphate-buffered saline after perfusion with NaCl. The ipsilateral DRG was extracted and post-fixed in 4% paraformaldehyde for 3 h at 4°C before being serially transferred to 15 and 30% sucrose for dehydration. Finally, the DRGs were also stored in a −80°C freezer for immunofluorescence experiments.

### Immunofluorescence Staining

Dorsal root ganglions were sliced at a thickness of 14 μm. The slices were blocked with 5% normal donkey serum in TBST (1% Tween-20) for 1 h at 37°C and then incubated with rabbit anti-PKCε (1:1000 in 5% normal donkey serum, Abcam, United States) or mouse anti-TRPV1 (1:1000 in 5% normal donkey serum, Abcam, United States) overnight at 4°C. The slices were then incubated in fluorescein AffiniPure donkey anti-rabbit (Alexa 647, Abcam, United States) or anti-mouse IgG (FITC, Jackson, United States) for 1 h at 37°C. Image of the expression of PKCε and TRPV1 in L4, L5, and L6 DRG was captured by using an A1R confocal microscope (Nikon, Tokyo, Japan). Slices that were used for the double labeling were incubated with a mixture of the antibodies. All the slices were stained with DAPI to identify the nuclei of all cell types in the ganglia.

The criteria were determined as follows. Firstly, 15 images were selected randomly. Then, two experimenters gave their own criteria (mainly the threshold) of the positive pixel. The average of the criteria was calculated and feedback to the two experimenters. Then, they negotiated and adjusted the criteria, including the shape, area, and fluorescence intensity (threshold). Finally, the threshold of positive pixel (gray value) was set and the positive area that shaping likes a circle was considered as a positive cell.

### Western Blot Analysis

The method has been described before (Du et al., 2020). Briefly, total protein from L4–L6 DRGs was extracted. RIPA Lysis Buffer (Beyotime, China) containing 1% PMSF (Beyotime, China) and a protease/phosphatase inhibitor cocktail (Applygen, China) was used to extract the protein. The protein concentration was detected by a BCA protein assay kit. Protein samples (20 μg) were separated on 5–10% SDS-PAGE gels and electrophoretically transferred to polyvinyl difluoride (PVDF) membranes (Bio-Rad, United States). The membranes were incubated with 5% low-fat milk in TBST for 1 h at room temperature, rabbit anti-PKCε (1:1000 in 5% normal goat serum, Abcam, United States), and anti-TRPV1 (1:1000 in 5% normal goat serum, Abcam, United States) for overnight at 4°C, and horseradish peroxidase (HRP)-conjugated goat anti-rabbit IgG (1:5000, Abcam, United States) for 1 h at room temperature. Rabbit anti-GAPDH (HRP conjugate) (1:1000, CST, United States) was used as the internal control. The membranes were developed with an ECL kit (Pierce, United States), and the signals were captured with an ImageQuant LAS 4000 system (EG, United States). The density of each band was measured using ImageQuant TL 7.0 analysis software (GE, United States). The mean expression level of the target proteins in animals from the normal or NS+PGE2 group was considered to be 1, and the relative expression level of the target proteins in all animals was normalized to the level of that group.

### Experimental Design and Groups

In this study, we tried to demonstrate the effect of EA on pain transition in three steps. First, we tried to determine the peripheral mechanism of pain transition using hyperalgesic priming model. We investigated changes in the MWT and TWL of the rats to ensure that we successfully established the model. In this part, the rats were randomly divided into three groups: the normal (only injection NS) (*n* = 18), NS+PGE2 (PGE2 injected following NS) (*n* = 18), and Car+PGE2 groups (PGE2 injected following Car) (*n* = 18). The expression levels of PKCε and TRPV1 were also investigated to identify the possible PKCε pathway. Then, we selectively inhibited the expression of PKCε or blocked the function of TRPV1 to show that they are both involved in PGE2 induced hyperalgesia. In this part, the rats were divided into three groups: the Car+PGE2 (*n* = 12), Car+PGE2+vehicle (*n* = 12), and Car+PGE2+PKCεV1-2 (Car+PGE2 rats that injection PKCεV1-2 before PGE2) (*n* = 12) groups or the Car+PGE2 (*n* = 12), Car+PGE2+vehicle (*n* = 12), and Car+PGE2+CPZ (Car+PGE2 rats that injection CPZ 48 h after PGE2) (*n* = 12) groups. In additions, the effect of PKCεV1-2 and CPZ on PKCε and TRPV1 was investigated to show the upstream and downstream relationship between PKCε and TRPV1. Finally, we used the selective PKCε activation peptide ψεRACK to replace PGE2 to demonstrate that the PKCε–TRPV1 pathway plays an important role in the transition from acute to chronic pain. In this part, the rats were randomly divided into two groups, the Car+PGE2 (*n* = 12) and Car+ψεRACK (ψεRACK was injected following Car) (*n* = 12) group. We also investigated the differences in the MWT, the TWL, and PKCε and TRPV1 expression between the groups in this part.

Second, we investigated the effect of EA on pain transition. We investigated the effect of EA on the MWT, the TWL, and PKCε and TRPV1 expression to show that EA not only produced a temporary analgesic effect but also regulated the transition from acute to chronic pain. In this part, the rats were randomly divided into four groups: the NS+PGE2 (*n* = 12), Car+PGE2 (*n* = 12), Car+PGE2+EA (Car+PGE2 rats that were treated by EA stimulation) (*n* = 12), and Car+PGE2+sham EA (Car+PGE2 rats that were given sham EA stimulation) (*n* = 12) groups. Then, we used ψεRACK to replace PGE2 to block the effect of EA to demonstrate the effect of EA on pain transition by a pharmacological method. In this part, the rats were randomly divided into three groups: the NS+PGE2 (*n* = 12), Car+ψεRACK (*n* = 12), and Car+ψεRACK+EA (Car+ψεRACK rats that were treated by EA stimulation) (*n* = 12) groups. The MWT, the TWL, and PKCε and TRPV1 expression were also investigated in this part. Then, we ip.l injected morphine was used to simulate the analgesic effect of EA and observed morphine’s effect on PKCε and TRPV1 expression. In this part, the rats were randomly divided into two groups: the Car+PGE2 (*n* = 6) and Car+PGE2+morphine (Car+PGE2 rats that were treated by morphine after PGE2 injection) (*n* = 6) groups.

Finally, we tried to prevent the transition from acute to chronic pain by applying EA stimulation before chronic pain was induced. In this part, the rats were randomly divided into three groups: the NS+PGE2 (*n* = 7), Car+PGE2 (*n* = 7), and Car+PGE2+EA II (Car+PGE2 rats that the EA was only given during the period between the first and second injection) (*n* = 7) groups. The effect of EA II on the MWT and the expression of PKCε and TRPV1 were tested in this part.

### Statistics

All data are presented as the mean ± SEM of *n* independent observations. Statistical comparisons were made using SPSS 19.0 statistical software. Student’s *t*-test was used to compare two independent samples, whereas one-way analysis of variance (ANOVA) followed by Bonferroni’s multiple comparison tests was used to compare three or more samples. For behavioral testing, the data were analyzed using two-way ANOVA with between-subjects factors followed by Bonferroni’s multiple comparison tests. The density of specific bands on the Western blots was normalized to the density of the normal or NS+PGE2 group. *P* < 0.05 was considered statistically significant.

## Results

### Changes in the MWT and TWL in the Hyperalgesic Priming Model

**FIGURE 1:**
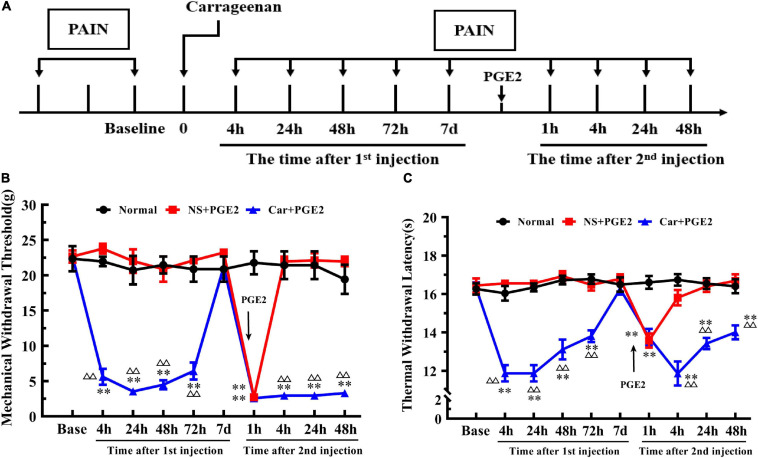
Carrageenan injection followed by PGE2 injection produces chronic mechanical and thermal pain. The protocol of modeling **(A)**. The mechanical withdrawal threshold (MWT) **(B)** and thermal withdrawal latency (TWL) **(C)** of animals that received carrageenan and PGE2 injections. *n* = 6. **Compared with the normal group, *P* < 0.01; ^△△^ compared with the NS+PGE2 group, *P* < 0.01.

The time of Car and PGE2 injection and of the pain threshold testing was shown in Figure 1A. We first investigated changes in the MWT of Car+PGE2 rats to ensure that the model was successfully established (Figure 1B). Two-way ANOVA was conducted to compare the effect of time on the MWT in the normal, NS+PGE2, and Car+PGE2 groups. The results revealed that there was a significant difference over time (*P* < 0.01, *F*_(2.085, 31.27)_ = 63.74) and between groups (*P* < 0.01, *F*_(2, 15)_ = 56.69) and there was also a significant interaction effect between time points and groups (*P* < 0.01, *F*_(18, 137)_ = 42.27). The *post hoc* Bonferroni test indicated that Car injection followed by PGE2 injection decreased the MWT in the rats (*P* < 0.01). However, PGE2 injection failed to change the MWT of rats compared with that of the normal group (*P* > 0.05). The results also showed that the MWT of NS+PGE2 and Car+PGE2 at 7 days after the first injection was not different from the normal group (*P* > 0.05).

One-way ANOVA for independent samples determined that Car intraplantar injection significantly decreased the MWT of Car+PGE2 rats between 4 and 72 h after the first injection (*P* < 0.01, *F*_4 *h*_ = 133.45, *F*_24 *h*_ = 45.50, *F*_48 *h*_ = 54.96, *F*_72 *h*_ = 45.76). The MWT of the Car+PGE2 rats group then recovered to the original level without any other treatment 7 days after the first injection (*P* > 0.05, *F* = 0.76). Then, PGE2, as an allogenic substance and inflammatory mediator, significantly decreased the MWT of the NS+PGE2 and Car+PGE2 groups 1 h after the second injection (*P* < 0.01, *F* = 130.98). There was little difference between these two groups in the MWT at that time point (*P* > 0.05). The MWT of both the NS+PGE2 and Car+PGE2 groups was lower than that of the normal group (*P* < 0.01). The MWT of the NS+PGE2 group quickly recovered to the normal level 4 h after PGE2 injection (*P* > 0.05, *F* = 79.27). However, the MWT of the Car+PGE2 group was lower than that of the normal and NS+PGE2 groups between 4 and 48 h (*P* < 0.01, *F*_4 *h*_ = 79.27, *F*_24 *h*_ = 70.74, *F*_48 *h*_ = 64.27). The changes in the MWT were similar to those that were previously described predecessors, which indicated that we successfully established the hyperalgesic priming model.

Then, we observed changes in the TWL (Figure 1C). The results of two-way ANOVA revealed that there was a significant difference over time (*P* < 0.01, *F*_(4.36, 65.4)_ = 11.00) and between groups (*P* < 0.01, *F*_(2, 15)_ = 167.70) and there was also a significant interaction between time and groups (*P* < 0.01, *F*_(18, 135)_ = 10.41). The *post hoc* Bonferroni test indicated that Car injection followed by PGE2 injection decreased the TWL of the Car+PGE2 rats (*P* < 0.01). And PGE2 injection failed to change the TWL of the rats compared with that of the normal group (*P* > 0.05). The results also showed that the TWL of Car+PGE2 group was not different from the normal group TWL 7 days after the first injection (*P* > 0.05).

Then, we compared the TWL of the different groups at each time point by one-way ANOVA. Car intraplantar injection decreased the TWL of the Car+PGE2 group between 4 and 72 h after the first injection (*P* < 0.01, *F*_4 *h*_ = 54.38, *F*_24 *h*_ = 81.34, *F*_48 *h*_ = 35.88, *F*_72 *h*_ = 32.95). Then, the TWL of the Car+PGE2 group recovered to the average level and did not differ from that of the normal and NS+PGE2 groups (*P* > 0.05, *F* = 0.75). The TWL of the NS+PGE2 and Car+PGE2 groups declined 1 h after PGE2 injection and was significantly lower than that of the normal group (*P* < 0.01, *F* = 19.62). The TWL of the NS+PGE2 group quickly recovered to the original level 4 h after the second injection and was not different from that of the normal group (*P* > 0.05, *F* = 31.16). However, the TWL of the Car+PGE2 group was continually declined 4 h after PGE2 injection and was significantly lower than that of the NS+PGE2 and normal group (*P* < 0.01). The TWL of the Car+PGE2 group was partly increased 24 h and 48 h after PGE2 injection but remained lower than that of the normal and NS+PGE2 groups (*P* < 0.01, *F*_24 *h*_ = 37.35, *F*_48 *h*_ = 17.29).

### PKCε Is Involved in Changes in the MWT and TWL

**FIGURE 2:**
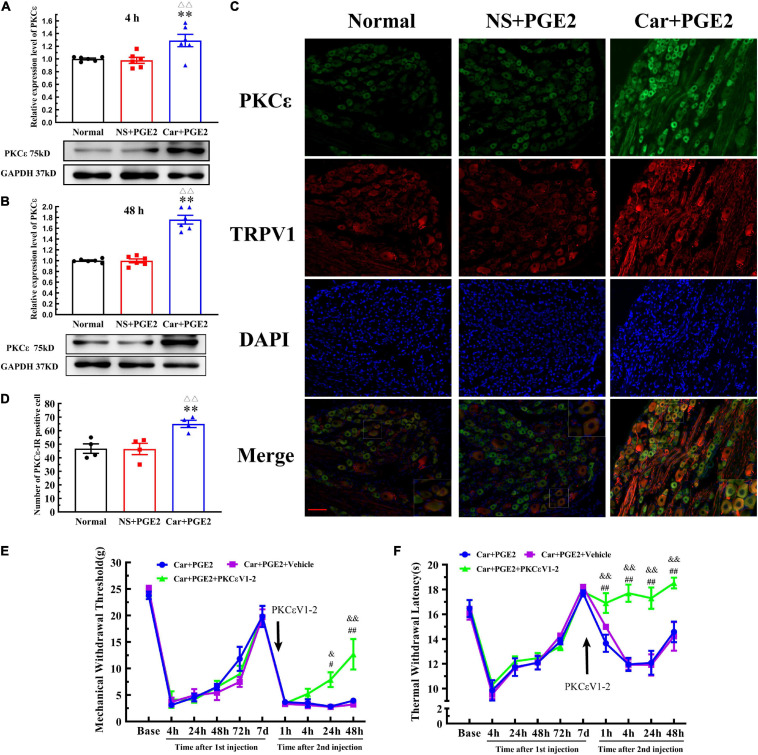
Protein kinase C epsilon (PKCε) in the dorsal root ganglion (DRG) plays a pivotal role in the transition from acute to chronic pain. **(A)** The quantification of the Western blot results and a representative Western blot showing PKCε protein isolated from the DRG 4 h after PGE2 injection. **(B)** The quantification of the Western blots results and a representative Western blot showing PKCε protein isolated from the DRG 48 h after PGE2 injection. **(C)** PKCε and TRPV1 staining in the peripheral nervous system 48 h after PGE2 injection. Scale bar 100 μm. **(D)** The quantification of PKCε–IR positive neurons. Mechanical **(E)** and thermal **(F)** responses of hyperalgesia model animals that received PKCεV1-2 injection. *n* = 6. **Compared with the normal group, *P* < 0.01; ^△△^ compared with the NS+PGE2 group, *P* < 0.01; ^#,##^ compared with the Car+PGE2 group, *P* < 0.05 and *P* < 0.01, ^&,&&^ compared with the Car+PGE2+vehicle group, *P* < 0.05, *P* < 0.01.

Because previous studies have suggested that PKCε in lumbar DRGs plays a pivotal role in the transition from acute to chronic pain, we tested whether PKCε expression level is changed in L4–L6 DRGs. The Western blotting results showed that the expression level of PKCε in L4–L6 DRGs in the Car+PGE2 group was higher than that in the normal and NS+PGE2 groups 4 and 48 h after the second injection (Figures 2A,B) (*P* < 0.01, *F*_4 *h*__(2, 15)_ = 7.71, *F*_48 *h(2, 15)*_ = 72.72). The expression level of PKCε in lumbar DRGs of the NS+PGE2 group was not different from that in the normal group at the same time point (*P* > 0.05). The immunofluorescence results showed that the number of PKCε–IR neurons in the L4–L6 DRGs in the Car+PGE2 group was higher than that in the normal and NS+PGE2 groups 48 h after PGE2 injection (Figures 2C,D) (*P* < 0.01, *F*_(2,9)_ = 9.30). There was little difference in the number of PKCε–IR neurons in the lumbar DRGs between the normal and NS+PGE2 groups (*P* > 0.05).

Then, we tested whether PGE2 injection following Car injection produced chronic pain via PKCε activation (Figure 2E). The selective PKCε inhibitor PKCεV1-2 was intraplantar injected 5 min before PGE2 injection. Two-way repeated measures ANOVA revealed a significant difference over time (*P* < 0.01, *F*_(2.714, 40.71)_ = 168.8), but not between different groups (*P* > 0.05, *F*_(2, 15)_ = 1.87). There was a significant interactive effect between time and groups (*P* < 0.01, *F*_(18, 135)_ = 3.97).

As shown in Figure 2E, PKCεV1-2 can only regulate the MWT of Car+PGE2 rats after the second injection. One-way ANOVA was used to analyze the MWT of the rats at each time point after the second injection. There was no significant difference between Car+PGE2 and Car+PGE2+PKCεV1-2 group in MWT 4 h after PGE2 injection (Figure 2E) (*P* > 0.05, *F* = 2.150). The MWT of the Car+PGE2+PKCεV1-2 group gradually increased from 24 to 48 h after the second injection and was significantly higher than that of the Car+PGE2 group (*P* < 0.01, *F*_24 *h*_ = 12.63, *F*_48 *h*_ = 9.91). However, the intraplantar injection of the vehicle of PKCεV1-2 did not produce any effect on the MWT of the Car+PGE2 rats (*P* > 0.05).

We also investigated whether PKCε is involved in the changes in the TWL in the hyperalgesic priming model (Figure 2F). Similar to the MWT, repeated measures ANOVA indicated that there was a significant difference over time (*P* < 0.01, *F*_(4.301, 64.52)_ = 50.25) and between the different groups (*P* < 0.01, *F*_(2, 15)_ = 24.29). There was a significant interaction between time and group (*P* < 0.01, *F*_(18, 135)_ = 5.86). The *post hoc* Bonferroni test indicated that PKCεV1-2 increased the TWL of the Car+PGE2 rats (*P* < 0.01). However, the vehicle of PKCεV1-2 failed to change the TWL of the Car+PGE2 rats (*P* > 0.05).

The results of one-way ANOVA test followed by Bonferroni’s multiple comparison tests revealed that PKCεV1-2 significantly increased the TWL from 4 to 48 h after PGE2 injection and that this level was higher than that of the Car+PGE2 rats (Figure 2F) (*P* < 0.01, *F*_4 *h*_ = 35.30, *F*_24 *h*_ = 11.68, *F*_48 *h*_ = 7.70). Furthermore, PKCεV1-2 also significantly increased the TWL 1 h after PGE2 injection (*P* < 0.01, *F* = 6.763). The intraplantar injection of the vehicle of PKCεV1-2 did not produce any effect on the TWL of the Car+PGE2 rats (*P* > 0.05).

### TRPV1 Is Involved in Changes in the MWT and TWL

**FIGURE 3:**
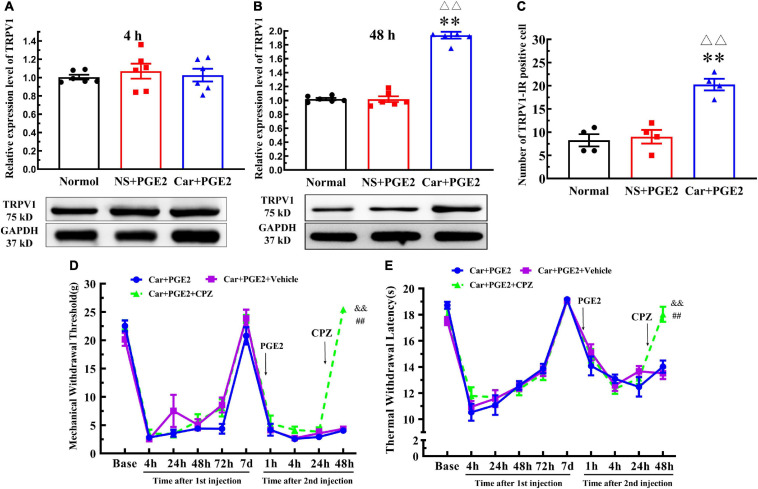
Transient receptor potential vanilloid 1 (TRPV1) in the DRG contributed to the chronic mechanical and thermal pain induced by carrageenan and PGE2 injections. **(A)** The quantification of the Western blot results and a representative Western blot showing TRPV1 protein isolated from the DRG 4 h after PGE2 injection. **(B)** The quantification of the Western blot results and a representative Western blot showing TRPV1 protein isolated from the DRG 48 h after PGE2 injection. **(C)** The quantification of TRPV1–IR positive neurons. Mechanical **(D)** and thermal **(E)** responses of hyperalgesia model animals that received capsazepine (CPZ) injection. *n* = 6. **Compared with the normal group, *P* < 0.01; ^△△^ compared with the NS+PGE2 group, *P* < 0.01; ^##^ compared with the Car+PGE2 group, *P* < 0.01; ^&&^ compared with the Car+PGE2+vehicle group, *P* < 0.01.

Protein kinase C epsilon is a signal molecule and could produce hyperalgesia by regulating the function of its downstream proteins. We tested the expression level of TRPV1, one of the most important downstream protein of PKCε, in the lumbar DRGs. The injection of PGE2 following Car injection significantly increased the protein level of TRPV1 in L4–L6 DRGs 48 h after the second injection (*P* < 0.01, *F*_(2, 15)_ = 192.1), but not 4 h after the second injection (*P* > 0.05, *F*_(2, 15)_ = 0.27) (Figures 3A,B). The number of TRPV1–IR-positive neurons was also higher than that in the normal group 48 h after PGE2 injection (Figures 2C, 3C) (*P* < 0.01, *F*_(2, 9)_ = 28.49). However, only PGE2 injection failed to promote the protein level of TRPV1 and the number of TRPV1–IR-positive neurons in L4–L6 DRGs (*P* > 0.05). Additionally, the co-localized of PKCε and TRPV1 can be found in L4–L6 DRGs, in the normal rats, NS+PGE2 rats, and Car+PGE2 rats, as shown in Figure 2C. The PGE2 injection following Car significantly up-regulated the proportion of TRPV1 labeled cells that expressing PKCε (Supplementary Figure 1A) *(P* < 0.01, *F*_(2, 9)_ = 24.25). However, only injection of PGE2 did not change the proportion of co-localized neurons.

Then, we investigated whether TRPV1 was involved in the change in the MWT and TWL in the hyperalgesic priming model by using CPZ. Because CPZ only regulated the MWT and TWL 48 h after the second injection, we only used one-way ANOVA to analyze the difference between the groups 48 h after PGE2 injection. The results indicated that CPZ significantly increased the MWT of the Car+PGE2 rats 48 h after PGE2 injection (Figure 3D) (*P* < 0.01, *F* = _(2, 15)_525.0). The TWL results were similar to the MWT results. CPZ significantly increased the TWL of the Car+PGE2 rats 48 h after PGE2 injection (Figure 3E) (*P* < 0.01, *F*_(2, 15)_ = 24.34). For the CPZ may also affect the function of some Ca^2+^ ion channel, the AMG9810 was also used to investigate the role of TRPV1 played. The results were similar to the CPZ results (Supplementary Figure 2) *(P* < 0.01, *F*_48 *hMWT(2, 15)*_ = 44.15, *F*_48 *hTWL(2, 15)*_ = 19.74).

### PKCε Promotes TRPV1 Expression in Lumbar DRGs

**FIGURE 4:**
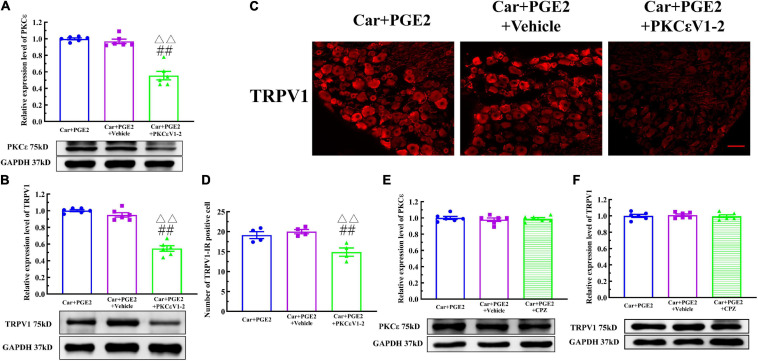
Protein kinase C epsilon (PKCε) regulates TRPV1 expression in the DRG. **(A)** The quantification of the Western blot results and a representative Western blot showing inhibited PKCε protein isolated from the DRG 48 h after PGE2 injection. **(B)** The quantification of the Western blot results and a representative Western blot showing decreased TRPV1 protein isolated from the DRG 48 h after PGE2 injection. **(C)** TRPV1 staining in the peripheral nervous system 48 h after PGE2 injection. Scale bar 100 μm. **(D)** The quantification of TRPV1–IR positive neurons. **(E)** The quantification of the Western blot results and a representative Western blot showing PKCε protein isolated from the DRG 4 h after PGE2 injection. **(F)** The quantification of the Western blot results and a representative Western blot showing TRPV1 protein isolated from DRG 48 h after PGE2 injection. ^△△^ Compared with the NS+PGE2 group, *P* < 0.01; ^##^ compared with the Car+PGE2 group, *P* < 0.01.

Because PKCε and TRPV1 are observed in the same neurons, we further investigated whether PKCε regulated the expression of TRPV1 in lumbar DRGs after PGE2 injection. First, we observed the effect of the inhibition of PKCε on the expression level of TRPV1. PKCεV1-2 significantly inhibited the expression of PKCε and downregulated the TRPV1 protein level in L4–L6 DRGs (Figures 4A,B) (*P* < 0.01, *F*_*PKC*ε (2, 15)_ = 52.55, *F*_*TRPV1(2, 15)*_ = 94.97). The vehicle of PKCεV1-2 did not affect the expression levels of PKCε and TRPV1 (*P* > 0.05). In addition, the immunofluorescence results showed that the number of TRPV1–IR neurons in the Car+PGE2+PKCεV1-2 group was significantly lower than that in the Car+PGE2 group and Car+PGE2+Vehicle group (Figures 4C,D) (*P* < 0.01, *F*_(2, 15)_ = 103.7). Additionally, we investigated whether a TRPV1 antagonist regulates the expression level of PKCε and TRPV1 in lumbar DRGs. The results of Western blot analysis showed that CPZ did not affect the expression levels of PKCε or TRPV1 in lumbar DRGs (Figures 4E,F) (*P* > 0.05, *F*_*PKC*ε (2, 15)_ = 0.34, *F*_*TRPV1*(2,15)_ = 0.13).

**FIGURE 5:**
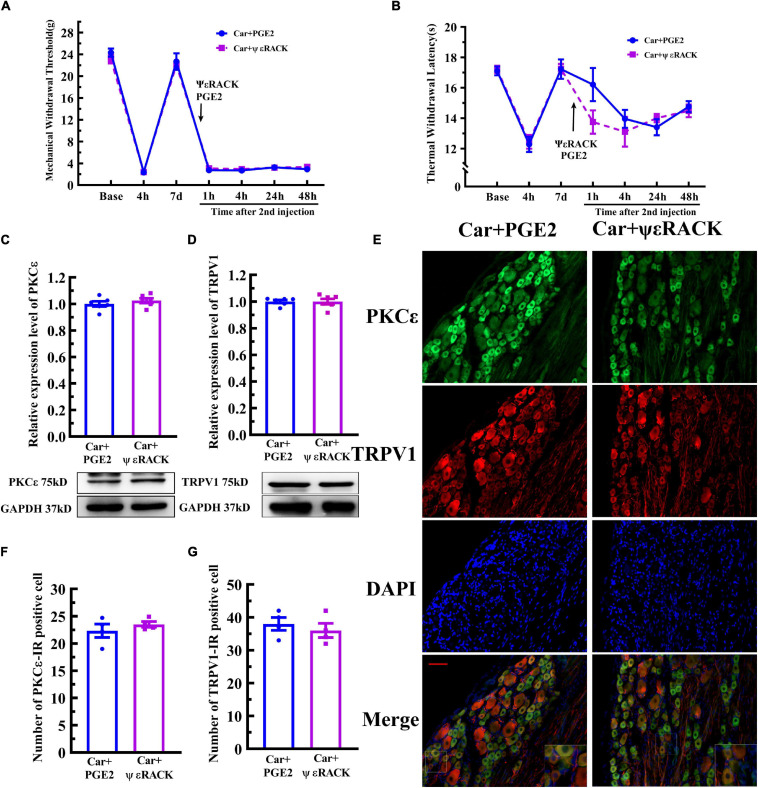
Activation of PKCε contributes to the transition from acute to chronic pain and TRPV1 expression. Mechanical **(A)** and thermal **(B)** responses of animals to the injection of a special PKCε activator. *n* = 6. The quantification of the Western blot results and a representative Western blot showing PKCε **(C)** and TRPV1 **(D)** protein isolated from the DRG 48 h after PGE2 and ψεRACK injection. **(E)** PKCε and TRPV1 staining in the peripheral nervous system 48 h after PGE2 and ψεRACK injection. Scale bar 100 μm. The quantification of PKCε–IR positive **(F)** and TRPV1–IR positive **(G)** neurons.

Then, ψεRACK, a selective agonist peptide of PKCε, was used instead of PGE2. The injection of ψεRACK, similar to that of PGE2, produced a long-lasting decrease in the MWT and TWL (Figures 5A,B) (*P* > 0.05, *F*_*MWT*__(1, 10)_ = 0.07, *F*_*TWL*__(1,10)_ = 0.52, *F* value of group factor). The Western blot results showed that there was little difference between the Car+PGE2 and Car+ψεRACK groups (Figure 5C) (*P* > 0.05, *t*_10_ = 0.917), which indicated that ψεRACK injection, like PGE2 injection, promoted the expression of PKCε in lumbar DRGs. ψεRACK injection also upregulated the TRPV1 protein level in L4–L6 DRGs (Figure 5D) (*P* > 0.05, *t*_10_ = 0.028). The number of PKCε–IR and TRPV1–IR cells in L4–L6 DRGs in the Car+PGE2 group was similar to that in the Car+ψεRACK group, and there was no significant difference (Figures 5E,F) (*P* > 0.05, *t*_6 *PKC*ε_ = 0.50, *t*_6 *TRPV1*_ = 0.69). The results of immunofluorescence also showed that ψεRACK-induced PKCε and TRPV1 expression were co-localized in the same neurons in L4–L6 DRGs (Figure 5G). Furthermore, ψεRACK significantly increased the number of TRPV1 labeled cells that expressed PKCε just as the effect of PGE2 produced (Supplementary Figure 1B) (*P* > 0.05, *t*_6_ = 0.45).

### EA Regulates Changes in the MWT and TWL in the Hyperalgesic Priming Model

**FIGURE 6:**
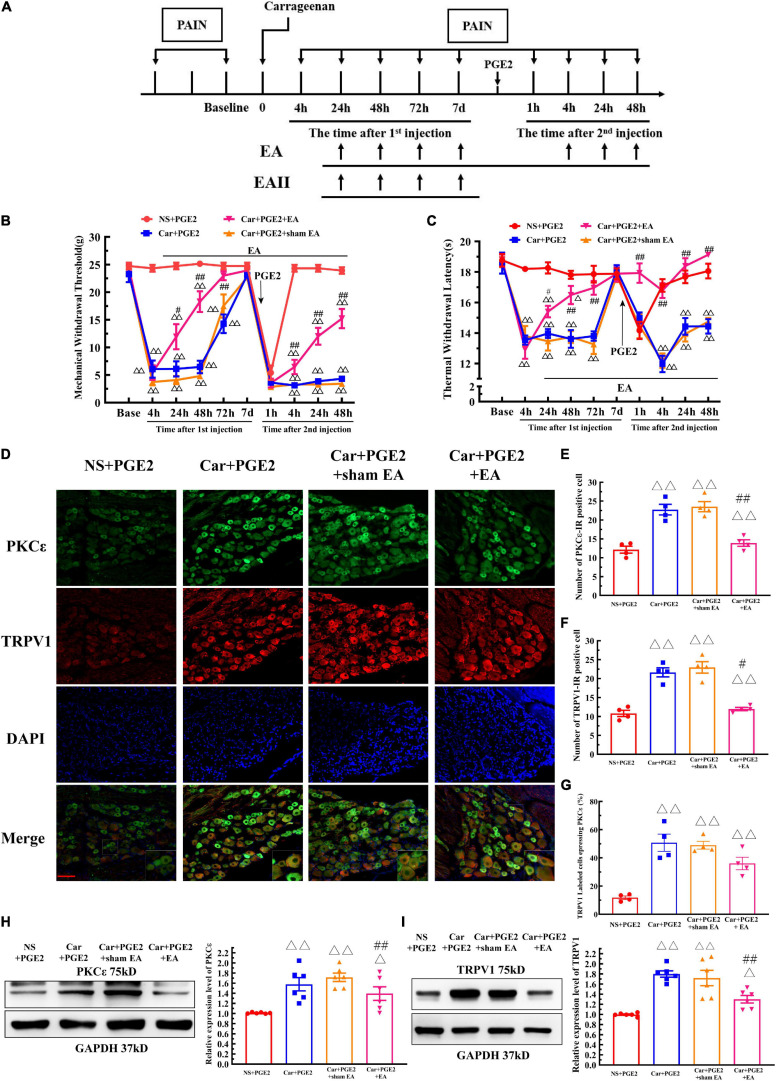
Electroacupuncture (EA) regulates the transition from acute to chronic pain and the PKCε activation and TRPV1 expression in the DRG. The protocol of EA administration **(A)**. Mechanical **(B)** and thermal **(C)** responses of hyperalgesic priming model animals to EA and sham EA administration. *n* = 5–6. **(D)** PKCε and TRPV1 staining in the peripheral nervous system 48 h after PGE2 injection. Scale bar 100 μm. The quantification of PKCε–IR positive **(E)** and TRPV1–IR positive **(F)** neurons. The quantification of the ratio of TRPV1 labeled cells expressing PKCε **(G)**. The quantification of the Western blot results and a representative Western blot showing PKCε **(H)** and TRPV1 **(I)** protein isolated from the DRG 48 h after PGE2 injection. ^△^, ^△△^ Compared with the NS+PGE2 group, *P* < 0.05, *P* < 0.01; ^#,##^ compared with the Car+PGE2 group, *P* < 0.05, *P* < 0.01.

Electroacupuncture was administered to regulate changes in the MWT and TWL in hyperalgesic priming model rats (Figure 6A), and the results are shown in Figures 6B,C. The results of two-way ANOVA indicated that there was a significant difference over time (*P* < 0.01, *F*_*MWT(3.48, 94.07)*_ = 230.0, *F*_*TWL(6.32, 126.4)*_ = 32.95) and between the different groups (*P* < 0.01, *F*_*MWT(3, 27)*_ = 97.18, *F*_*TWL(3, 20)*_ = 115.4). There was a significant interaction between time and group (*P* < 0.01, *F*_*MWT(24, 243)*_ = 28.38, *F*_*TWL(27, 180)*_ = 8.56) for both the MWT and TWL. The *post hoc* Bonferroni test indicated that PGE2 injection following Car injection significantly decreased the MWT and TWL (*P* < 0.01). EA treatment partly increased the MWT (*P* < 0.01), but the MWT was still lower than that of NS+PGE2 rats (*P* < 0.01). However, EA administration significantly restored the TWL, and the TWL of the Car+PGE2+EA group was not different from that of the NS+PGE2 group (*P* > 0.05). Sham EA did not affect the effect of PGE2 and Car on the MWT and TWL (*P* > 0.05).

The results of one-way ANOVA showed that EA significantly regulated the decrease in the MWT induced by Car injection from 24 to 72 h after the first injection (*P* < 0.05, *F*_24 *h*_ = 41.20, *F*_48 *h*_ = 66.38, *F*_72 *h*_ = 12.40). However, EA failed to modulate the decrease in the MTW 1 h after PGE2 injection (*P* > 0.05, *F* = 1.77). Then, EA significantly increased the MWT, and the MWT of the Car+PGE2+EA group was higher than that of the Car+PGE2 group 4, 24, and 48 h after PGE2 injection (*P* < 0.01, *F*_4 *h*_ = 166.57, *F*_24 *h*_ = 123.33, *F*_48 *h*_ = 93.42). However, the MWT of the Car+PGE2+EA group was still significantly lower than that of the NS+PGE2 group at the final time point observed (*P* < 0.01). Additionally, sham EA did not affect the MWT of the Car+PGE2 rats at any time point (*P* > 0.05).

The effect of EA on the TWL of the priming model rat was also statistically analyzed by one-way ANOVA. Similar to the MWT results, EA significantly upregulated the TWL of the Car injection rats. The TWL of the Car+PGE2+EA group was considerably higher than that of the Car+PGE2 group from 24 to 72 h after the first injection (*P* < 0.05, *F*_24 *h*_ = 25.18, *F*_48 *h*_ = 18.96, *F*_72 *h*_ = 20.59). There was little difference between the Car+PGE2+EA and NS+PGE2 groups 72 h after Car injection (*P* > 0.05). PGE2 administration following Car injection produced a significant reduction in the TWL of the rats. EA stimulation upregulated the TWL of the Car+PGE2 rats from 4 to 48 h after the second injection (*P* < 0.01, *F*_4 *h*_ = 34.13, *F*_24 *h*_ = 20.69, *F*_48 *h*_ = 40.91). The TWL of the Car+PGE2+EA group was not different from that of the NS+PGE2 group (*P* > 0.05) and was much higher than that of the Car+PGE2 group from 4 to 48 h after PGE2 injection (*P* < 0.01). Finally, sham EA failed to regulate the TWL changes regardless of whether they were induced by Car or PGE2 (*P* > 0.05).

Electroacupuncture was also administrated to the bilateral unrelated acupoints “Quchi” (LI 11, radial proximal anterior joint) and “Waiguan” (TE 5, 3 mm above the wrist) on hyperalgesic priming rats. The results of two-way ANOVA indicated that EA on unrelated acupoints would not produce analgesic effect on hyperalgesic priming rats (Supplementary Figure 3) (*P* > 0.05, *F*_*MWT*__(2, 12)_ = 118.5, *F*_*TWL*__(2, 12)_ = 19.97, *F* value of group factor).

### EA Regulates the High Expression of PKCε and TRPV1 in Lumbar DRGs in a Hyperalgesic Priming Model

Because EA affected the MWT and TWL of hyperalgesic priming model rats, we further investigated whether EA regulated the expression of PKCε and TRPV1 in lumbar L4–L6 DRGs. As shown in Figure 6D, TRPV1 and PKC ε were co-localized in the same neurons in lumbar DRGs. Consistent with previous results, PGE2 injection following Car injection upregulated the number of PKCε–IR and TRPV1–IR neurons of in lumbar DRGs 48 h after the second injection, as well as the number of TRPV1 labeled cells expressing PKCε (Figures 6E–G) (*P* < 0.01, *F*_*PKC*ε (3, 12)_ = 53.71, *F*_*TRPV1(3, 12)*_ = 66.12, *F*_*CO(3, 12)*_ = 19.76). The number of PKCε–IR and TRPV1–IR neurons in the Car+PGE2+EA group was significantly lower than that in the Car+PGE2 group (*P* < 0.05), which indicated that EA regulated the expression of PKCε and TRPV1 in lumbar DRGs. However, EA only partly decreased the number of TRPV1 labeled cell that expressing PKCε (Figure 6G) (*P* > 0.05). The Western blot results were similar. The expression levels of PKCε and TPRV1 in the Car+PGE2 group were higher than those in the NS+PGE2 group 48 h after PGE2 injection (Figures 6H,I) (*P* < 0.01, *F*_*PKC*ε (3, 20)_ = 8.87, *F*_*TRPV1(3, 20)*_ = 17.40), consistent with the results shown in Figures 2, 3. Furthermore, the expression levels of PKCε and TRPV1 in the Car+PGE2+EA group were lower than those in the Car+PGE2 group and higher than those in the NS+PGE2 group (*P* < 0.01). Finally, there was little difference in the expression levels of PKCε and TRPV1 between the Car+PGE2 and Car+PGE2+sham EA groups (*P* > 0.05).

**FIGURE 7:**
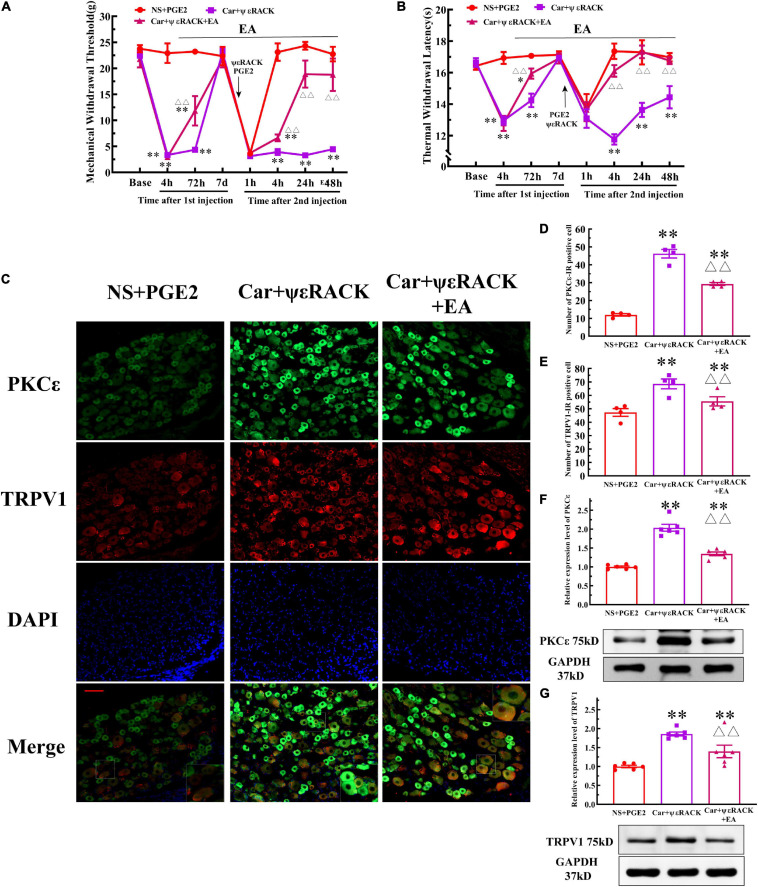
Electroacupuncture (EA) regulates the transition from acute to chronic pain induced by ψεRACK injection. Mechanical **(A)** and thermal **(B)** responses of Car/ψεRACK-injected animals to EA administration. *n* = 6. **(C)** PKCε and TRPV1 staining in the peripheral nervous system 48 h after ψεRACK injection. Scale bar, 100 μm. The quantification of the PKCε–IR positive **(D)** and TRPV1–IR positive **(E)** neurons. The quantification of the Western blot results and a representative Western blot showing PKCε **(F)** and TRPV1 **(G)** protein isolated from the DRG 48 h after ψεRACK injection. *, **Compared with the NS+PGE2 group, *P* < 0.05, *P* < 0.01; ^△△^ compared with the ψεRACK group, *P* < 0.01.

Then, we observed whether EA regulates the hyperalgesia induced by ψεRACK and the higher expression of PKCε and TRPV1. ψεRACK injection induced both a persistent reduction in the MWT and TWL (Figures 7A,B) (*P* < 0.01, *F*_*MWT(2, 15)*_ = 49.13, *F*_*TWL(2, 15)*_ = 110.5, *F* value of group factor) and the higher expression of PKCε and TRPV1 (Figure 7C–G) (*P* < 0.01). As shown in Figure 7, EA significantly increased the MWT and TWL of the rats injected with ψεRACK. The one-way ANOVA results indicated that these effects were observed from 4 to 48 h after the second injection (Figures 7A,B) (*P* < 0.01, *F*_*MWT4 h*_ = 88.69, *F*_*MWT24 h*_ = 47.65, *F*_*MWT48 h*_ = 22.81, *F*_*TWL4 h*_ = 53.42, *F*_*TWL24 h*_ = 14.04, *F*_*TWL48 h*_ = 9.50). Immunofluorescence showed that the number of PKCε–IR and TRPV1–IR neurons in the Car+ψεRACK+EA group was lower than that in the Car+ψεRACK (use ψεRACK instead of PGE2 for inducing hyperalgesic priming) group (Figures 7C–E) (*P* < 0.01, *F*_*PKC*ε (2, 9)_ = 129.3, *F*_*TRPV1(2, 9)*_ = 10.37), but was still higher than that in the NS+PGE2 group (*P* < 0.01). The protein expression levels of PKCε and TRPV1 in the DRG of the Car+ψεRACK+EA group were also lower than those of the Car+ψεRACK group (Figures 7F,G) (*P* < 0.01, *F*_*PKC*ε (2, 15)_ = 73.57, *F*_*TRPV1(2, 15)*_ = 17.83) but were still higher than those of the NS+PGE2 group (*P* < 0.01).

**FIGURE 8:**
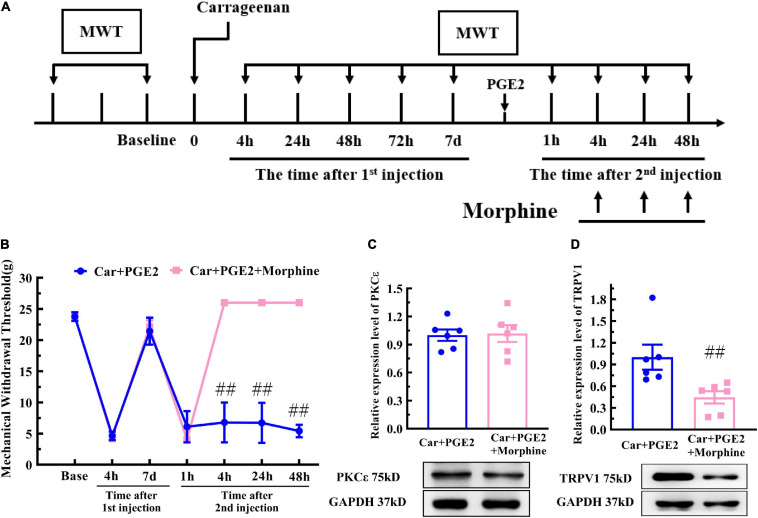
Morphine improves the MWT of hyperalgesic priming rats and inhibits the TRPV1 expression in L4–L6 DRG, but not PKCε. The protocol of morphine administration **(A)**. **(B)** Mechanical responses of hyperalgesic priming model animals to morphine administration. *n* = 6. Western blot showing PKCε **(C)** and TRPV1 **(D)** protein isolated from the DRG 48 h after PGE2 injection. ^##^Compared with the Car+PGE2 group, *P* < 0.01.

Electroacupuncture can produce an analgesic effect on various pain through the endogenous opioid system. Morphine has been used to simulate the analgesic effect of EA on the hyperalgesic priming model (Figure 8A). The analgesic effect of morphine has been shown in Figure 8B. Two-way ANOVA reveal a significant difference over time (*P* < 0.01, *F*_(1, 35)_ = 102.7) and between groups (*P* < 0.01, *F*_(6, 35)_ = 40.58). There was a significant interaction between time and group (*P* < 0.01, *F*_(6,35)_ = 25.35). The *post hoc* Bonferroni test indicated that morphine administered after PGE2 injection successfully regulates the decrease in the MWT (*P* < 0.01).

One-way ANOVA was used to compare the MWT at each time point after the second injection. Morphine injection significantly reversed the hyperalgesia induced by the PGE2 injection following Car injection at 4, 24, and 48 h after the second injection (*P* < 0.01, *t*_4 *h(*__10__)_ = 6.03, *t*_24 *h(10)*_ = 6.01, *t*_48 *h(10)*_ = 20.72). Then, we observed the effect of morphine injection on the PKCε and TRPV1 expression 48 h after the PGE2 injection. The results showed that morphine injection failed to regulate the higher expression level of PKCε in the L4–L6 DRG (Figure 8C) (*P* > 0.05, *t*_10_ = 0.16). However, morphine significantly decreased the expression level of TRPV1 in L4–L6 DRG (Figure 8D) (*P* < 0.01, *t*_10_ = 2.89).

### EA Partly Prevents the Transition of Pain and PKCε Expression

**FIGURE 9:**
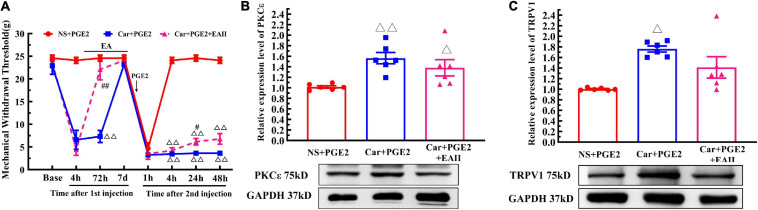
Electroacupuncture (EA) partly prevented the transition from acute to chronic pain. Mechanical **(A)** responses of hyperalgesia model animals to EA administration only after the first injection. *n* = 6. The quantification of the Western blot results and a representative Western blot showing PKCε **(B)** and TRPV1 **(C)** protein isolated from the DRG 48 h after PGE2 injection. ^△^, ^△△^ Compared with the NS+PGE2 group, *P* < 0.05, *P* < 0.01; ^#,##^ Compared with the Car+PGE2 group, *P* < 0.05, *P* < 0.01.

Finally, we examine whether EA can prevent the decrease in the MWT induced by PGE2 injection following Car injection if it is not given after the second injection (Figure 6A, EA II). The results are shown in Figure 9A. Two-way repeated measures ANOVA reveal a significant difference over time (*P* < 0.01, *F*_(4.25, 72.32)_ = 162.9) and between groups (*P* < 0.01, *F*_(2, 17)_ = 162.9). There was a significant interaction between time and group (*P* < 0.01, *F*_(14, 119)_ = 38.34). The *post hoc* Bonferroni test indicated that Car injection followed by PGE2 injection caused a decrease in the MWT of the rats (*P* < 0.01). EA administered only before PGE2 injection failed to regulate the decrease in the MWT (*P* > 0.05).

Then, we used one-way ANOVA to compare the MWT at each time point. The results indicated that EA raised the MWT 72 h after the first injection, and that the MWT of the Car+PGE2+EA II group was significantly higher than that of the Car+PGE2 group (*P* < 0.01, *F* = 26.84). The MWT of all the rats recovered to the level of the NS+PGE2 group 7 days after the first injection. There was little difference in the MWT among the three groups 1 h after PGE2 injection (*P* > 0.05, *F* = 0.51). The MWT of the NS+PGE2 groups was higher than that of the Car+PGE2 and Car+PGE2+EA II groups from 4 to 48 h after the second injection (*P* < 0.01, *F*_4 *h*_ = 394.76, *F*_24 *h*_ = 303.192, *F*_48 *h*_ = 179.87). The MWT of the EA II group was higher than that of the Car+PGE2 group, but only 24 h after the second injection (*P* < 0.05). There was little difference between the MWT of the EA II and Car+PGE2 groups 48 h after injection.

Because EA administration before PGE2 injection partly restores the MWT of the Car+PGE2 rats, we wanted to determine whether it can regulate the expression of PKCε and TRPV1 in lumbar DRGs. The Western blot results are shown in Figure 9B. PKCε expression in the NS+PGE2 group was much lower than that in the Car+PGE2 (*P* < 0.01, *F*_(2, 15)_ = 6.54) and Car+PGE2+EAII groups (*P* < 0.05). EA partly inhibited PKCε expression in L4–L6 DRGs, but the PKCε expression level in this group was not different from that in the Car+PGE2 group (*P* > 0.05). We also tested the expression level of TRPV1, as shown in Figure 9C. TRPV1 expression in the NS+PGE2 group was lower than that in the Car+PGE2 group (*P* < 0.05, *F*_(2, 15)_ = 9.66) and was not significantly different from that in the EAII group (*P* > 0.05). However, there was only a trend for the TRPV1 expression level in the Car+PGE2+EAII group to be lower than that in the Car+PGE2 group (*P* > 0.05).

## Discussion

Although many cases of pain are resolved through a variety of treatment options, more than 20% of the population in the United States and 30% of the population in Europe suffers from chronic pain for years (Gaskin and Richard, 2012; Breivik et al., 2013). In general, pain that persists beyond the time needed for tissue injury recovery is considered chronic pain. However, the causes of the continuation of pain after tissue damage recovery are not yet understood. Furthermore, there are few effective treatment strategies for preventing the transition from acute to chronic pain (Heinricher, 2016; Pozek et al., 2016). The hyperalgesic priming model has been used to study the mechanism of the transition from acute to chronic pain and to identify the treatment strategies (Kim et al., 2016; Chen et al., 2018). Here, we use this model system to gain insight into whether EA can regulate the transition from acute to chronic pain and the mechanism. The main conclusion we reached from our experiments is that EA can reverse pain transition in hyperalgesic priming model rats by inhibiting the PKCε and TRPV1 expression in the peripheral nervous system and that this effect may be partly due to its interference in the prime state.

Electroacupuncture is a modern way of applying acupuncture and is widely used for alleviating various types of pain, such as inflammatory pain, neuropathological pain, and cancer pain (Fang et al., 2018; Liang et al., 2018, 2019). Previous studies have demonstrated that EA can relieve both acute and chronic pain (Seo et al., 2017; Chai et al., 2018). Our team had shown that EA can regulate the PKCε expression in the DRG of hyperalgesic priming rats in the previous study (Wang et al., 2020). However, it is not clear whether EA is able to prevent the transition from acute to chronic pain. Because EA can produce an analgesic effect on chronic pain through various mechanisms, it is challenging to observe the preventive effect of EA on pain transition directly. In the current study, we demonstrated that EA could prevent pain transition through three steps.

First, we showed that the PKCε (which molecule plays a pivotal in the pain transition (Ferrari et al., 2014; Kandasamy and Price, 2015)-dependent TRPV1 activation is involved in the transition from acute to chronic pain. Besides PKCε, other PKC isoforms, such as PKA and PKCγ, have been suggested to be involved in pain sensitization (Warner et al., 2017; Alba-Delgado et al., 2018). Previous studies demonstrated that PKA is involved in the opioid receptor induced hyperalgesia priming but not acute inflammation (Araldi et al., 2016). The intradermal injection of PKCεV1-2 prevents the prolonged mechanical hyperalgesia induced by PGE2 in a Car-induced priming model (Aley et al., 2000; Hassouna et al., 2004). The transient attenuation of PKCε expression in the periphery by antisense oligodeoxynucleotides is able to terminate chronic pain states (Parada et al., 2003). These studies suggest that peripheral PKCε plays a role in the initiation and maintenance of long-lasting hyperalgesia in a Car-induced priming model. Furthermore, the activation of the PKCε–TRPV1 pathway has been demonstrated to be involved in the development of hyperalgesia and neuropathic pain (Malek et al., 2015; Gu et al., 2018). Here, we showed that the intradermal injection of PGE2 following Car injection also promoted the expression level of TRPV1. In addition, the intradermal administration of a TRPV1 antagonist alleviated hyperalgesia (Figure 3). Furthermore, previous study demonstrated that the neuron excitability will raise accompanying the high expression of TRPV1, which can induce strong cation influx (Wu et al., 2013). That maybe the reason that produces the hyperalgesia on the model rats. In this study, the attenuation of PKCε expression in the periphery by a selective inhibitor not only reversed the chronic pain state but also decreased TRPV1 expression. In addition, immunofluorescence showed that the co-localization of PKCε and TRPV1 increased in the model rats. Additionally, the intradermal injection of ψεRACK, a PKCε-specific activator peptide (Karouzaki et al., 2019), instead of PGE2 produced an increase in the expression of TRPV1 in L4–L6 DRGs and induced chronic pain. All these results indicate that PGE2 produced long-lasting hyperalgesia by activating the PKCε–TRPV1 pathway in the peripheral nervous system in a Car-induced priming model.

In contrast to the previous studies, we synchronously observed the thermal pain threshold of a hyperalgesic priming model. Thermal and mechanical stimuli are commonly used in chronic pain studies in animals and humans and cause qualitatively different pain sensations. Previous studies have shown that different pain systems induce different characteristics of chronic pain. For instance, a reduction of mechanical and thermal pain thresholds will occur in the chronic inflammatory pain model induced by CFA (Li et al., 2019). However, there is only a pain response to mechanical stimuli in the neuropathic pain model induced by SNI surgery (Shields et al., 2003). EA is able to regulate both mechanical and thermal pain sensitization (Liao et al., 2017). However, whether the hyperalgesic priming model induces a thermal sensitization is not clear. We showed that PGE2 produced long-time thermal hyperalgesia after transient inflammatory irritation (Figure 1). We further demonstrated that the PKCε–TRPV1 pathway in the peripheral nervous system not only contributed to mechanical sensation but was also involved in thermal hyperalgesia by using pharmacological methods. This is consistent with the function of TRPV1 in mechanical and thermal pain. Furthermore, calmodulin-dependent protein kinase II (CaMKII) and extracellular regulatory protein kinase (ERK) are both downstream signaling molecules of PKCε (Ferrari et al., 2013; Zisopoulou et al., 2013) but are upstream of TRPV1 (Ma et al., 2017). And they are both involved in the pain transition in hyperalgesic priming model (Ferrari et al., 2014). So, the PKCε–CaMKII/ERK–TRPV1 signaling pathway may contribute to the pain transition in DRG.

Second, we showed that EA can alleviate hyperalgesia and regulate the expression levels of PKCε and TRPV1 in the peripheral nervous system. Throughout the entire experiment, EA significantly increased the pain threshold of the model rats (Figure 6). EA not only recovered the mechanical and thermal pain induced by Car but also partly improved the mechanical hyperalgesia from 4 to 48 h after the second injection. Furthermore, the effect of EA on thermal hyperalgesia was much better than that on mechanical hyperalgesia. EA raised the thermal pain threshold to the original level at the 4 h after the second injection. This treatment effect was entirely beyond our expectations. We will further study the cause of these results. To show that EA interferes with the transition from acute to chronic pain, rather than just anesthesia, we examined the effect of EA on PKCε and TRPV1 expression. EA significantly regulated the higher expression of PKCε and TRPV1 in peripheral nervous system. For PKCε–TRPV1 pathway in peripheral nervous system is involved in the hyperalgesia in the Car-induced priming model, the analgesic effect of EA on the hyperalgesic priming model has a positive relationship with its inhibitory effect on the PKCε and TRPV1 expression. Furthermore, we used pharmacological experiments to test the effects of EA on PKCε and TRPV1 expression and hyperalgesia by selective activation of PKCε in peripheral nervous system. EA also regulated ψεRACK-induced hyperalgesia and the higher expression of PKCε and TRPV1 in L4–L6 DRGs. Moreover, morphine has been used to investigate whether EA regulates the pain transition via its analgesic effect. For morphine injection will induce the hyperalgesic priming type II, it has only been used after the PGE2 injection and the number of injections was controlled less than four times (Tumati et al., 2010; Araldi et al., 2016). Morphine significantly inhibited the expression of TRPV1 in L4–L6 DRG, just as previous report (Bao et al., 2015). However, although morphine produces a great analgesic effect on the hyperalgesic priming rats, it failed to inhibit the activation of PKCε in L4–L6 DRGs. These results indicated that the analgesic effects of EA and morphine on hyperalgesic priming rats have different mechanisms, especially on the PKCε activation or expression. However, PKCε is the key molecule of PGE2 induced chronic pain. So, we believed that EA not only produced analgesic effect on the model animal but also prevented the pain transition by inhibiting the PKCε expression. In addition, previous studies demonstrated that CaMKII and ERK were involved in the EA analgesia (Gu et al., 2020; Hu et al., 2020). So, we hypothesized that CaMKII and ERK may also be involved in the regulatory effect of EA on the pain transition according to the role they played in the pain transition and EA analgesia.

Third, we performed a time window experiment to observe the effect of EA on priming state. Previous studies divided the hyperalgesic priming model into two phases: the prime state, in which animals may not show significant pain abnormalities, but the nociceptive system responds to a normally subthreshold noxious stimulation with long-lasting hyperalgesia, and the hyperalgesic state, in which animals show significant pain abnormalities and the PKCε-dependent pathway is activated in the peripheral nerves system. Even if EA is given only in the prime state, it can still affect hyperalgesia, but the effect is significantly reduced. In this study, the statistical difference in behavioral performance was only observed 24 h after the second injection, but not 4 or 48 h after the second injection. Although PKCεV1-2 increased the MWT of the model rats 4 h after PGE2 injection, a significant difference was observed 24 h after the second injection (Figure 2E). Therefore, we believe that there may still be some acute pain induced by PGE2 4 h after the second injection and that interferes with the effect of EA. Furthermore, the average value of the MWT in the EA II group was higher than that in the Car+PGE2 group. However, the standard error of the MWT in the EA II group was also increased 48 h after second injection. There might be two reasons for this. On the one hand, due to individual differences, the regulatory effect of EA in certain animals is diminished. On the other hand, this might be just a statistical error. We will further study it and attempt to improve the effect of EA. In addition, only giving EA in the prime state still induce a certain regulatory trend in the expression level of PKCε and TRPV1 in L4–L6 DRGs. Therefore, we believe the EA administered only in the prime state may partly affect the ability of PGE2 to activate the PKCε-dependent pathway and thus produce a mild analgesic effect. Furthermore, all the results also indicate that the interference of EA in pain transition is mainly due to the regulatory effect after PKCε activation, rather than interference in the prime state.

Above all, we believe that EA can prevent the transition from acute to chronic pain by regulating the PKCε and TRPV1 expression in the peripheral nervous system. And it makes EA to be more widely used in clinical analgesia. For some patients with acute pain, EA analgesia may prevent the occurrence of pain transition along with its analgesic effect. More importantly, even if it is nearly impossible to determine the time point of the transition from acute to chronic pain in the clinic practice for chronic pain patients, EA can still be used to prevent the pain transition with slight risks. The worst case is that EA alleviates constant pain but fails to prevent pain transition in case EA was not applied during the appropriate period. Ideally, EA may limit the development of chronic pain. On the other hand, EA can be combined with other analgesic drugs. Treatment programs may achieve better analgesia while EA prevents pain transition.

## Conclusion

Electroacupuncture regulates the transition from acute to chronic pain by inhibiting the PKCε and TRPV1 expression in the peripheral nervous system.

## Data Availability Statement

The original contributions presented in the study are included in the article/Supplementary Material, further inquiries can be directed to the corresponding author/s.

## Ethics Statement

The animal study was reviewed and approved by Experimental Animal Center Affiliated Zhejiang Chinese Medical University.

## Author Contributions

JD and XJ: conceptualization. JuF: data curation. JuF, JiF, JZ, and JD: funding acquisition. JuF, SW, XS, YL, YJ, and BL: investigation. SW, HS, and XH: methodology. JiF: writing–original draft. JD: writing–review and editing. All authors contributed to the article and approved the submitted version.

## Conflict of Interest

The authors declare that the research was conducted in the absence of any commercial or financial relationships that could be construed as a potential conflict of interest.

## Abbreviations

DRG: dorsal root ganglion
MWT: mechanical withdrawal threshold
TWL: thermal withdrawal latency
PKC ε: protein kinase C epsilon
PGE2: prostaglandin E2
EA: electroacupuncture.
