# Supplementary material for: Electroacupuncture Regulates Pain Transition Through Inhibiting PKCε and TRPV1 Expression in Dorsal Root Ganglion
Source: Front Neurosci. 2021 Jul 20;15:685715. doi: 10.3389/fnins.2021.685715 (PMC8329384; doi:10.3389/fnins.2021.685715)
Supplement: Supplementary file 1 [file Table_1.DOCX]

Supplementary Material

Electroacupuncture Regulates Pain Transition through Inhibiting PKCε Activation and TRPV1 Expression in DRG

Junfan Fang, Sisi Wang, Jie Zhou, Xiaomei Shao, Haiju Sun, Yi Liang, Xiaofeng He,

Yongliang Jiang, Boyi Liu, Xiaomin Jin, Jianqiao Fang, and Junying Du

# Supplementary Figure 1


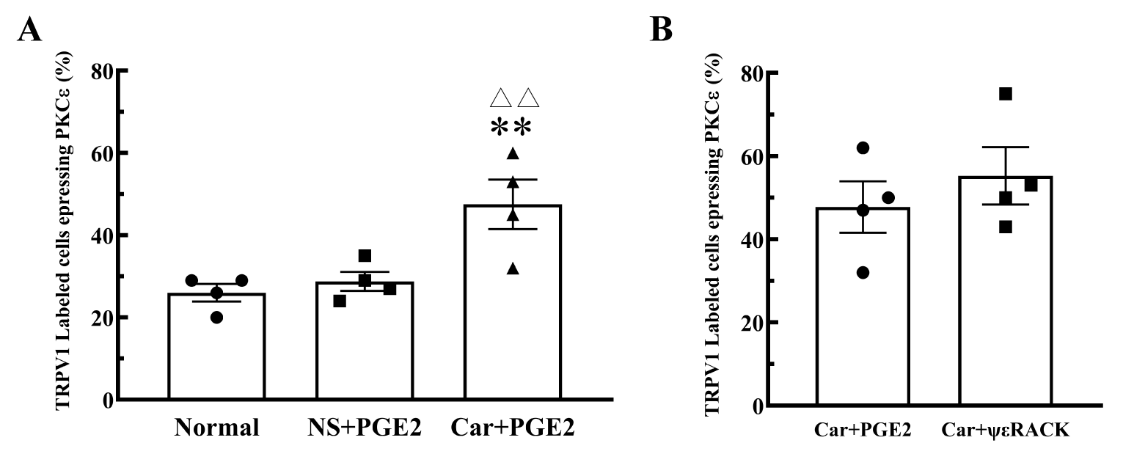


**Figure 1 The ratio of TRPV1 labeled cells expressing PKCε.** PGE2 injection following carrageenan injection up-regulate the ratio of TRPV1 labeled cells expressing PKCε, as shown in Figure A. The ψεRACK could increase the ratio of TRPV1 labeled cells expressing PKCε as the PGE2 effect (B).

# Supple**mentary Figure 2**


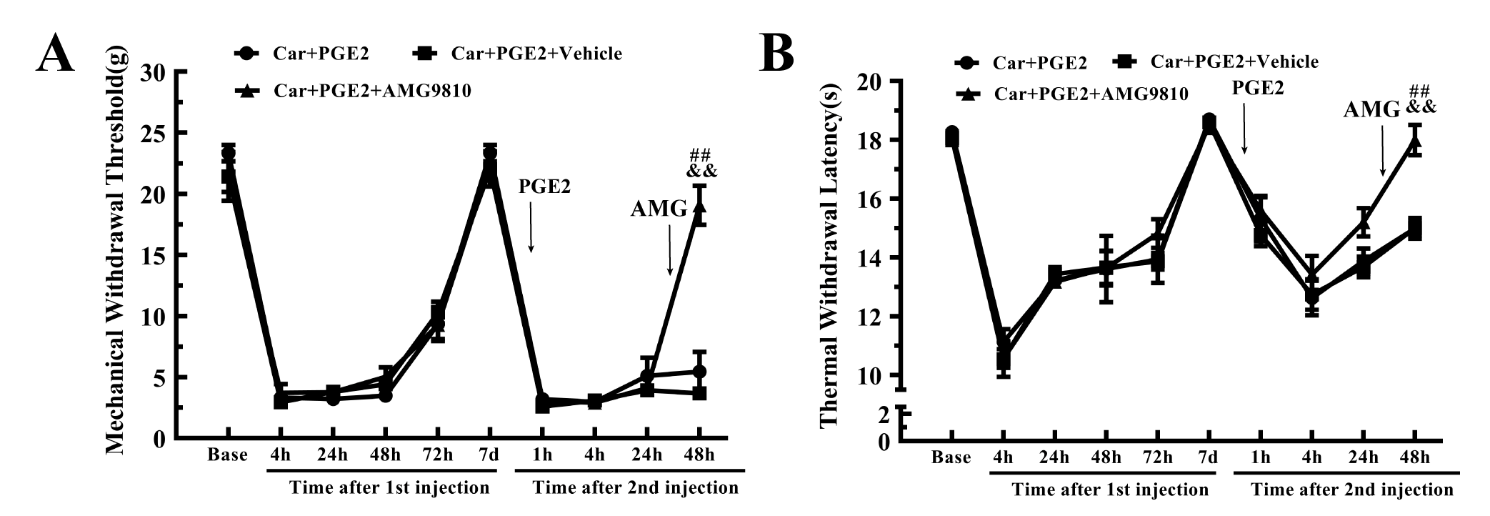


**Figure 2 AMG9810 significantly reversed the pain induced by PGE2 injection following carrageenan.** Mechanical (A) and thermal (B) responses of hyperalgesia model animals that received AMG9810 injection. n=6. ## compared with Car+PGE2 group, *P*<0.01; && compared with Car+PGE2+vehicle group, *P*<0.01.

# Supplementary Figure 3


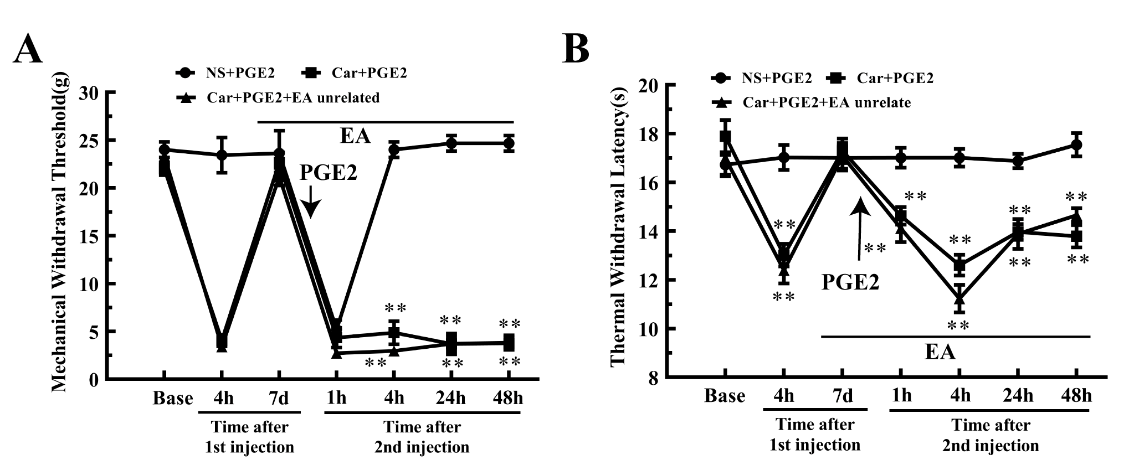


**Figure 3 EA on unrelated acupoints does not affect the hyperalgesia priming.** EA stimulated on bilateral unrelated acupoints “Quchi” (LI 11, radial proximal anterior joint) and “Waiguan” (TE 5, 3mm above the wrist) does not regulate the MWT (A) or TWL(B) of hyperalgesic priming rats on any time points. n=6. ** Compared with NS+PGE2 group, *P*<0.01*.*
